# Neuroplastin deletion in glutamatergic neurons impairs selective brain functions and calcium regulation: implication for cognitive deterioration

**DOI:** 10.1038/s41598-017-07839-9

**Published:** 2017-08-04

**Authors:** Rodrigo Herrera-Molina, Kristina Mlinac-Jerkovic, Katarina Ilic, Franziska Stöber, Sampath Kumar Vemula, Mauricio Sandoval, Natasa Jovanov Milosevic, Goran Simic, Karl-Heinz Smalla, Jürgen Goldschmidt, Svjetlana Kalanj Bognar, Dirk Montag

**Affiliations:** 1 Department of Neurochemistry and Molecular Biology, Leibniz Institute for Neurobiology, Magdeburg, Germany; 2 Croatian Institute for Brain Research, School of Medicine, University of Zagreb, Zagreb, Croatia; 3 Department of Systems Physiology; Special Laboratories, Leibniz Institute for Neurobiology, Magdeburg, Germany; 4 Department of Molecular Biology Techniques, Leibniz Institute for Neurobiology, Magdeburg, Germany; 5 Neurogenetics, Leibniz Institute for Neurobiology, Magdeburg, Germany

## Abstract

The cell adhesion molecule neuroplastin (Np) is a novel candidate to influence human intelligence. Np-deficient mice display complex cognitive deficits and reduced levels of Plasma Membrane Ca^2+^ ATPases (PMCAs), an essential regulator of the intracellular Ca^2+^ concentration ([iCa^2+^]) and neuronal activity. We show abundant expression and conserved cellular and molecular features of Np in glutamatergic neurons in human hippocampal-cortical pathways as characterized for the rodent brain. In *Nptn* ^*lox/loxEmx1Cre*^ mice, glutamatergic neuron-selective Np ablation resulted in behavioral deficits indicating hippocampal, striatal, and sensorimotor dysfunction paralleled by highly altered activities in hippocampal CA1 area, sensorimotor cortex layers I-III/IV, and the striatal sensorimotor domain detected by single-photon emission computed tomography. Altered hippocampal and cortical activities correlated with reduction of distinct PMCA paralogs in *Nptn* ^*lox/loxEmx1Cre*^ mice and increased [iCa^2+^] in cultured mutant neurons. Human and rodent Np enhanced the post-transcriptional expression of and co-localized with PMCA paralogs in the plasma membrane of transfected cells. Our results indicate Np as essential for PMCA expression in glutamatergic neurons allowing proper [iCa^2+^] regulation and normal circuit activity. Neuron-type-specific Np ablation empowers the investigation of circuit-coded learning and memory and identification of causal mechanisms leading to cognitive deterioration.

## Introduction

Learning and memory depend on balanced excitation and inhibition in neuronal networks interconnected *via* synapses^1–5^. Although the underlying molecular mechanisms are not fully understood, it is clear that cell adhesion molecules (CAMs) regulate decisive mechanisms to fine-tune neuronal communication^6–8^. The CAM neuroplastin^9^ (Np) regulates synaptic plasticity i.e. long-term potentiation^10–12^ and formation/stabilization of excitatory synapses^13, 14^ and balances the ratio of excitatory/inhibitory synapses^13^.

Among 50,000 gene variants analyzed, a promoter polymorphism in the human Np gene (*NPTN*) correlated with cortical thickness and cognitive capabilities in adolescents^15^. Abnormally increased Np expression is linked to schizophrenia^16^. Recently, new mouse models for the study of Np functions have been reported. Mice with loss of Np65 expression (one of the two Np isoforms, Np55 and Np65) display altered spatial performance and anxiety-like behaviors^17^. Other groups have reported that mice with polymorphic or truncated Np gene variants are deaf^14, 18^. In contrast *Nptn* ^−/−^ mice, completely devoid of Np expression, are not deaf but display loss of fear-conditioned associative learning, altered sensorimotor capabilities, complex swimming and diving behaviors as well as further deficits related to autism, depression, and schizophrenia^10^. Moreover, neuron-specific *Nptn* gene inactivation in adult *Nptn* ^*lox/loxPrpCreERT*^ mice triggered hippocampal and cortical alterations and induced retrograde amnesia of associative memories assigning a unique role to Np in cognitive functions^10^.

Although the distribution of neural CAMs such as N-CAM or L1 are similar in human, rat, and mouse hippocampus^19^, differences in hippocampal Np distribution between these species were reported^20^. Detailed expression, abundance, subcellular distribution or synaptic localization of the Np isoforms in the human brain have not been clarified. Thus, important information necessary to relate behavioral deficiencies in Np mouse models to specific human brain functions is missing.

In *Nptn* ^−/−^ and *Nptn* ^*lox/loxPrpCreERT*^ mice, PMCA expression levels are severely reduced^10^. These high-affinity Ca^2+^-pumps, but no other mechanisms, are capable to fully restore basal [iCa^2+^] in all eukaryotic cells^21, 22^. In neurons, PMCAs are key players in iCa^2+^-homeostasis essential for proper neuronal activity. Alternative splicing confers diversity in expression and localization to four PMCA paralogs (PMCA1–4)^23–28^. PMCA1 and 4 display ubiquitous expression in the mammalian brain with high levels in the hippocampal CA1 area and neocortical and piriform neurons^24–26^. PMCA2 is expressed in the hippocampus and specific PMCA2 isoforms are restricted to either GABAergic presynapses^27^ or glutamatergic spines^28^. PMCA3 is expressed in cortical regions with lower levels in the hippocampus^23^. Information on the specific contribution of PMCA paralogs to normal brain capabilities is scarce. Furthermore, despite alterations in the expression of PMCA paralogs and their association to impaired [iCa^2+^] handling in autism^29, 30^, schizophrenia^31^, Alzheimer’s disease^32, 33^, Niemann-Pick disease^34^, deafness^35^, and spinocerebellar ataxia^36, 37^, mechanisms leading to altered levels of PMCA paralogs remain largely unsolved.

Here we show that, similarly as reported for rodent, essential cellular and molecular features of Np are conserved in the human brain. Furthermore, human Np is also mainly expressed in glutamatergic neurons of hippocampal-cortical circuits. In mice, Np ablation in glutamatergic neurons impaired specific behaviors and activities in CA1 hippocampal area and in the cortex-striatum sensorimotor circuit. The differential loss of selective PMCA paralogs as direct consequence of Np ablation suggests the Np-PMCA interplay as a novel mechanism associated with brain area-specific cognitive functions.

## Results

**Figure 1:**
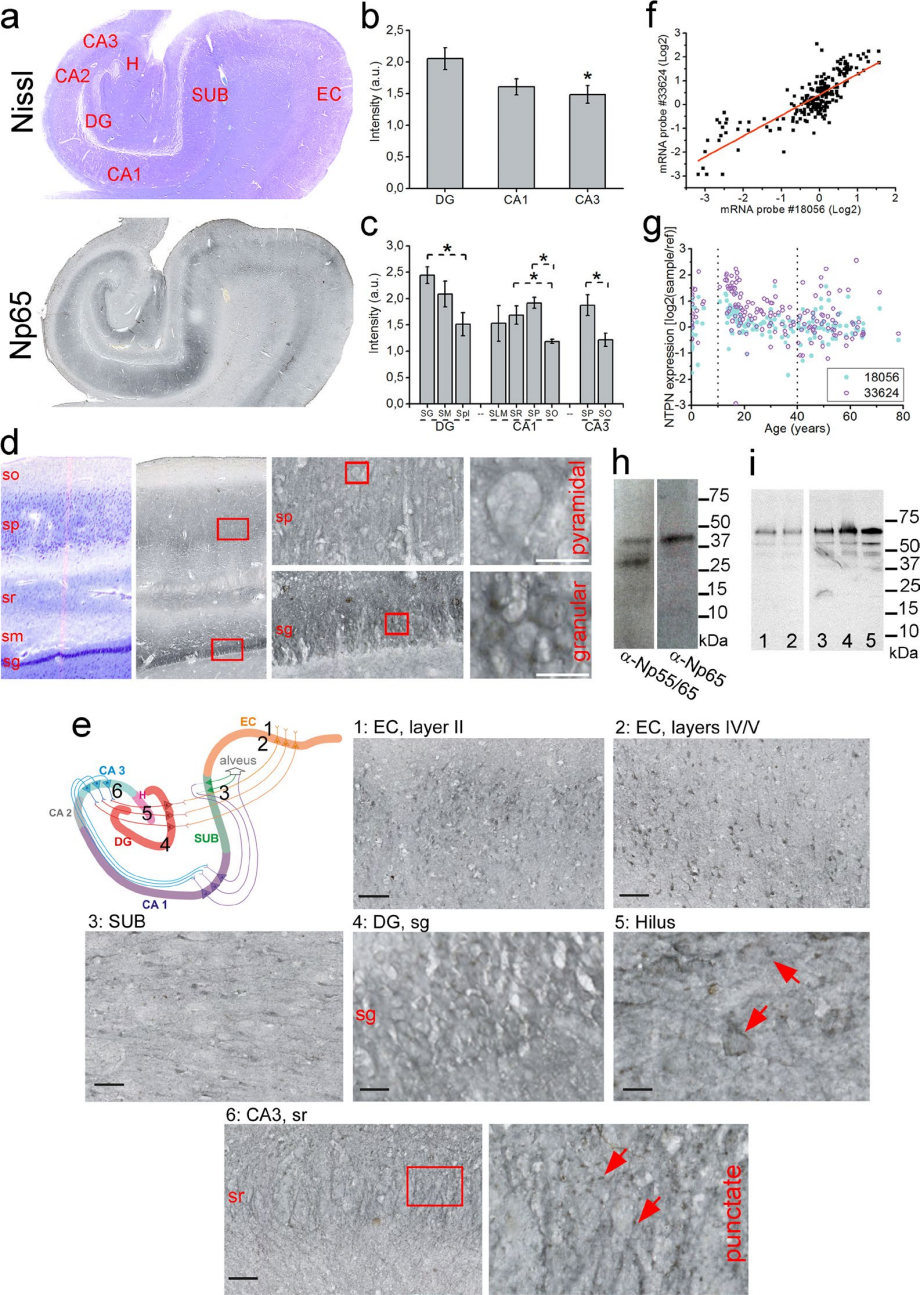
Localization, gene expression, and subcellular distribution of hNp. (**a**,**d**,**e**) Representative scans of Nissl staining and DAB-based detection of Np65 in human hippocampal-entorhinal cortex sections (**b**,**c**) Quantification of Np65 staining in hippocampal areas and sublayers (*P < 0.05, student t-test). (**d**) Magnification of CA1-DG area. Sequential close-ups are indicated with red frames. Scale bar = 20 µm. (**e**) Color-code drawing of the main glutamatergic pathways in the hippocampal-entorhinal cortex circuit. Numbers from 1 to 6 indicate the corresponding position of each Np65 scan. Scale bars in 1–3 and 6 = 100 µm, 4 and 5 = 20 µm. (**f**) mRNA expression correlation for Np65 (probe 33624, BrainCloud database)^55^ vs. Np55/65 (probe 18056) (red line: Pearson’s coefficient = 0.7988). (**g**) Np mRNA expression during embryonic, young-adult, and elderly stages (dotted lines). (**h**) Chemically deglycosylated homogenates from human cortex blotted with a pan-Np55/65 or an anti-Np65 antibody. (**i**) Np55/65 in subcellular fractions from human brain cortex. 1. total homogenate, 2. total membrane, 3. synaptic membrane, 4. synaptic junction, 5. post-synaptic density (PSD). A picture of the original blot is shown in Supplementary Fig. S1d. DG, dentate gyrus; CA cornu ammonis; H, hilus; SUB, subiculum; EC, entorhinal cortex; SG, stratum granulosum; SM, stratum moleculare; Spl, stratum plexiforme; SLM, stratum lacunosum-moleculare; SR, stratum radiatum; SP, stratum pyramidale; SO, stratum oriens.

Recently, Np has received much interest because of its correlation with cortical thickness and intelligence in adolescents^15, 38^. As Np expression has not been sufficiently characterized in human brain, we investigated the expression and molecular characteristics of human Np (hNp) further. First, preservation of hNp immunoreactivity, specificity of pan-Np55/65 and anti-Np65 antibodies and reliability of our immunohistochemistry procedures were demonstrated with hippocampal slices from 4 or 8 days post-mortem wild-type and *Nptn* ^−/−^ mice prepared under similar conditions as the human samples (Supplementary Fig. S1). Then, using Nissl staining, DAB-based detection and large-scale and high-resolution scanning (Fig. 1a), we analyzed hNp65 isoform immunoreactivity within the main areas/ layers of human hippocampal formation. hNp65 was mainly segregated to all major neuron-containing layers of the hippocampus –*stratum granulosum* and *stratum moleculare* of the dentate gyrus, the hilar part of the CA3 region, the pyramidal cell layer of CA1 and CA2-3 and the cellular layers of subiculum (Fig. 1a) as demonstrated by semi-automated analysis (Fig. 1b,c). Prominent hNp65 immunoreactivity is detected in densely packed apical dendrites of granular neurons and in *stratum moleculare* of DG. Presence of hNp65 is low in *stratum plexiforme* and within the hilar neuropil of DG, and we did not detect hNp65 in mossy fibers traversing the polymorphic layer of the DG (Fig. 1a,d). Also, it seems probable that some interneurons in the molecular layer of DG are hNp-positive as reported^20^. hNp65 expression was low and scattered throughout the hilar part of the CA3 region, but strong in cell bodies of glutamatergic pyramidal neurons and dendrites of *stratum radiatum* in CA1 while moderate in *strata pyramidale* of CA2-3 and subiculum (Fig. 1a–e). At the cellular level, hNp65 is very abundant at membranes of the cell body of granular and pyramidal neurons (Fig. 1d,e), dendrites, and in punctate structures within the neuropil (Fig. 1d,e). In the hilus, many hNp65-positive cells (Fig. 1e) largely resemble big multipolar calretinin-positive interneurons described previously^39^. In the entorhinal cortex, a prominent hNp65 localization is observed in glutamatergic pyramidal neurons in the layers II, IV and V (Fig. 1a,e) strikingly similar to the rat Np65 (rNp65) immunoreactivity^12^. In conclusion, unequivocally identified hNp65-positive glutamatergic neurons are granular neurons of DG, pyramidal neurons of CA1, CA2–3, subiculum, and layers II, IV, and V of the entorhinal cortex. Further direct visual inspection using a bright-field microscope confirmed that the hNp65 expression in major areas/ layers of the glutamatergic pathways within the entorhinal cortex and hippocampus (Supplementary Table 1) is very similar to its reported expression in mouse and rat^9, 12, 13, 20, 40^.

The *NPTN* gene is highly expressed in human striatum (see Supplementary Fig. S2 for Np protein expression), cortex and hippocampus^41^ (http://hbatlas.org/hbtd/images/wholeBrain/NPTN.pdf). Correlation analysis shows that hNp55/65 and hNp65 transcripts are tightly co-expressed in the prefrontal cortex of 0–80 year-old subjects (Fig. 1f). The highest hNp mRNA level occurred in 10–20 year-old subjects to decline afterwards with aging (P = 5.8^E-4^, two samples t tests, Fig. 1g). On the protein level, as described for rodent brain^40^, we detected two protein backbones of 28 and 40 KDa with a pan-Np55/65 antibody and only one of 40 KDa using an Np65-specific antibody in human brain homogenates after deglycosylation (Fig. 1h). Also, Np55/65- and Np65-associated immunoreactivity was co-localized and substantially similar in plasma membranes of human and mouse glutamatergic hippocampal neurons as observed using confocal microscopy (Supplementary Fig. S3). The hNp55/65 content in synaptic membranes, synaptic junctions, and postsynaptic densities (PSDs) was higher than in other subcellular fractions from human cortex (Fig. 1i) indicating relative enrichment of the human isoforms in glutamatergic synapses.

As human (Fig. 1 and Supplementary Fig. S1) and mouse^9, 12, 13, 20, 40^ Np55/65 share common molecular features and are expressed abundantly by hippocampal and cortical glutamatergic neurons, we generated *Nptn* ^*lox/loxEmx1Cre*^ mice to test whether Np deletion in these neuronal types affects selective behaviors. Importantly, hippocampal-cortical Np expression was eliminated specifically in glutamatergic but not in GABAergic neurons (Supplementary Fig. S4). *Nptn* ^*lox/loxEmx1Cre*^ mice displayed normal basic movement abilities and grip strength (Supplementary Fig. S5).

**Figure 2:**
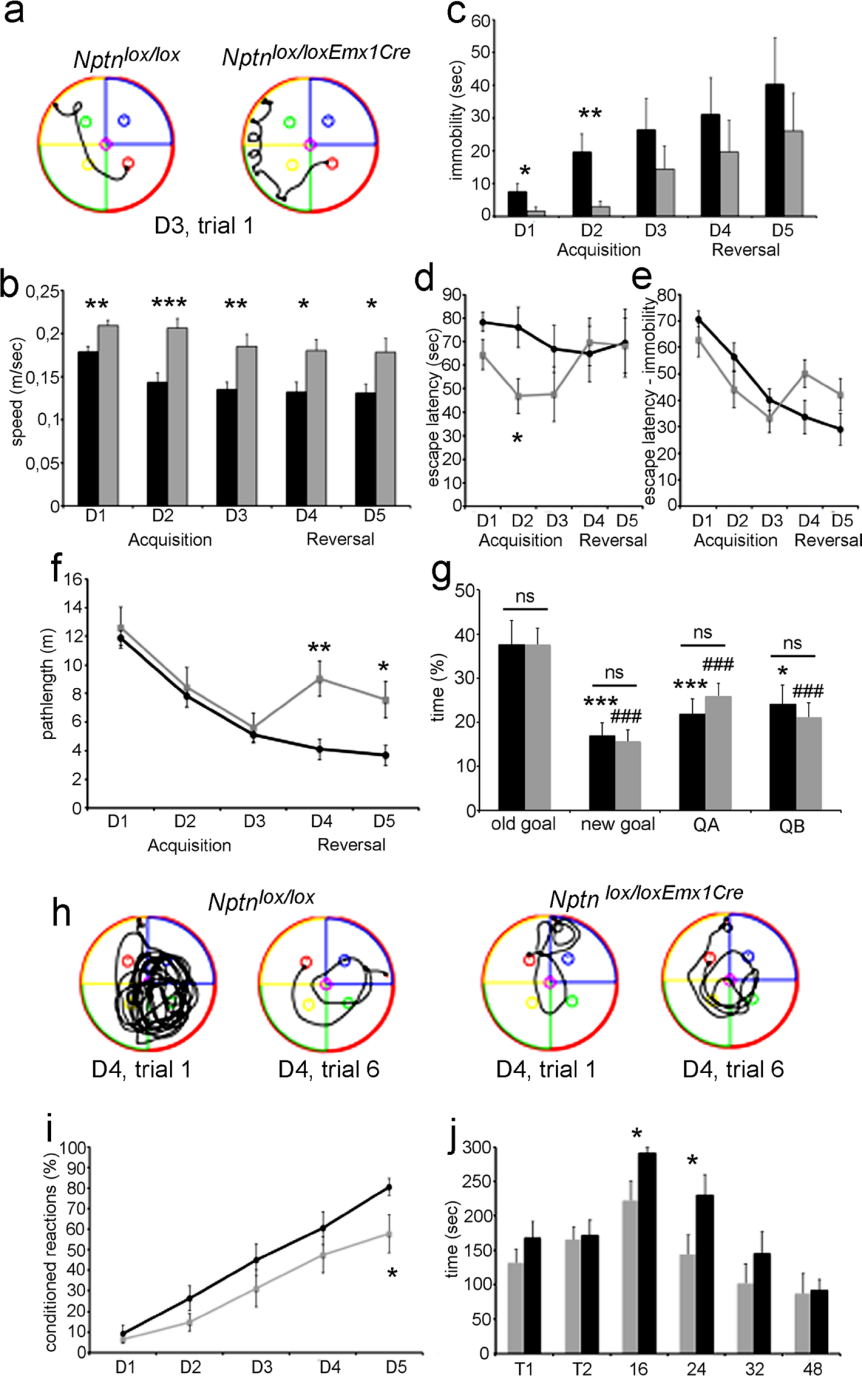
Behavioral deficits in *Nptn* ^*lox/loxEmx1Cre*^ mice. *Nptn* ^*lox/loxEmx1Cre*^ (n = 11, black bars and lines) and *Nptn* ^*lox/lox*^ (n = 12, grey) mice were examined (**a**–**h**) in the water maze, (**i**) in the shuttle box, (**j**) and on the rota-rod. (**a**) Representative examples of tracks recorded during acquisition (day3, 1st trial) showing frequent re-initiation of swimming by *Nptn* ^*lox/loxEmx1Cre*^ mice. (**b**) Average speed, (**c**) times of immobility, (**d**) escape latency, (**e**) escape latency corrected for times of immobility, and (**f**) path length are displayed. (**g**) Quadrant preference during the first 2 trials after platform reversal at day 4. QA, QB quadrants without platform. The old goal quadrant was significantly preferred compared to the other quadrants irrespective of the genotype of the mice. (**h**) Representative examples of tracks recorded immediately (day4, trial1) after platform reversal and after training (day4, trial6) for the new platform position. *Nptn* ^*lox/lox*^ mice initially persist on the old goal (trial1) and develop quickly a new spatial strategy (trial 6), whereas *Nptn* ^*lox/loxEmx1Cre*^ mice display a serial circular strategy. (**i**) The percentage of conditioned reactions in the shuttle box two-way active avoidance paradigm, (**j**) and the time staying on the rota-rod are displayed. Data are mean ± SEM. One-way ANOVA, one, two, and three asterisks indicate P < 0.05, P < 0.01, and P < 0.001, respectively, three # indicate P < 0.001.

In the Morris water maze, *Nptn* ^−/−^ mice show an aberrant diving behavior^10^ and pause swimming very frequently (data not shown) compromising the evaluation of spatial memory and strategies. Notably, *Nptn* ^*lox/loxEmx1Cre*^ mice did not dive but displayed frequent pausing during swimming as evidenced by the appearance of “knots” in the track recordings (Fig. 2a). Consistently, the average speed of *Nptn* ^*lox/loxEmx1Cre*^ mice was lower (Fig. 2b) (day 1, D1: F_(1,21)_ = 12.413, P = 0.002; D2: F_(1,21)_ = 16.683, P = 0.0005; D3: F_(1,21)_ = 8.674, P = 0.0077; D4: F_(1,21)_ = 7.181, P = 0.014; D5: F_(1,21)_ = 5.885, P = 0.0244) caused by longer lasting immobility (Fig. 2c) (D1: F_(1,21)_ = 4.968, P = 0.0369; D2: F_(1,21)_ = 8.783 P = 0.0074) resulting in longer escape latencies during acquisition (D2: F_(1,21)_ = 6.645, P = 0.0175; Fig. 2d) compared to control mice. When the escape latencies were corrected for times of immobility (Fig. 2e), *Nptn* ^*lox/loxEmx1Cre*^ and *Nptn* ^*lox/lox*^ mice showed the typical reduction in escape latency during acquisition at days 1–3 (Fig. 2e). In agreement, *Nptn* ^*lox/loxEmx1Cre*^ and *Nptn* ^*lox/lox*^ control mice showed similar reduction of the pathlength during acquisition (D1-D3; Fig. 2f). Furthermore, *Nptn* ^*lox/loxEmx1Cre*^ and *Nptn* ^*lox/lox*^ mice develop spatial memory as revealed by similar preference for the quadrant where the platform was located during acquisition (old goal) *vs*. the quadrant with the new platform position (new goal) or other quadrants (QA, QB) during the first two trials after platform reversal (Fig. 2g). However, reversal of the platform at D4-D5 expectedly prolonged only in *Nptn* ^*lox/lox*^ mice escape latency (Fig. 2e) and pathlength (Fig. 2f) (D4: F_(1,21)_ = 11.216, P = 0.003; D5: F_(1,21)_ = 6.456, P = 0.019), whereas *Nptn* ^*lox/loxEmx1Cre*^ mice were not perturbed at all. This lack of delay by platform reversal may indicate alternative non-spatial navigation by *Nptn* ^*lox/loxEmx1Cre*^ mice. Supportingly, the track recordings for day 4 (Fig. 2h) immediately after platform reversal indicate for *Nptn* ^*lox/lox*^ an initial persistence to the old platform position (trial 1) that changes into a more spatial strategy with further training for the new position (trial 6). In contrast, *Nptn* ^*lox/loxEmx1Cre*^ display after reversal a circular non-spatial strategy swimming equidistant to the wall indicating a hippocampal dysfunction related to building an allocentric representation of their environment required for a coordinated navigation. In conclusion, Np ablation in glutamatergic neurons did not interfere with formation of spatial memory but affected (a) the continuity of task execution with repetitive pause-swimming cycles indicative for altered striatum-dependent decision making and (b) the swimming strategy after platform reversal suggesting hippocampal deficits^42^.

*Nptn* ^−/−^ and induced *Nptn* ^*lox/loxPrpCreERT*^ mice are unable to learn associative fear conditioned tasks^10^. Here, we investigated whether Np deletion only in glutamatergic neurons is sufficient to impair this associative capacity. Using the two-way active avoidance shuttle-box paradigm, *Nptn* ^*lox/loxEmx1Cre*^ mice performed similar to *Nptn* ^*lox/lox*^ controls during training from D1 to D4, but slightly better at D5 (Fig. 2i) (F_(1,21)_ = 4.748, P = 0.0409), indicating that Np expression in glutamatergic neurons is not essential for this associative learning task. Using the rota-rod test (Fig. 2j), *Nptn* ^*lox/loxEmx1Cre*^ mice showed significantly better sensorimotor-coordination at 16 rpm (F_(1,21)_ = 5.149, P = 0.0339) and 24 rpm (F_(1,21)_ = 4.343, P = 0.0496) compared to *Nptn* ^*lox/lox*^ and consistent with the improved performance of *Nptn* ^*lox/loxPrpCreERT*^ mice^10^. Thus, Np ablation in glutamatergic neurons is sufficient to produce behavioral alterations related to cerebellar and/or motor cortex function.

**Figure 3:**
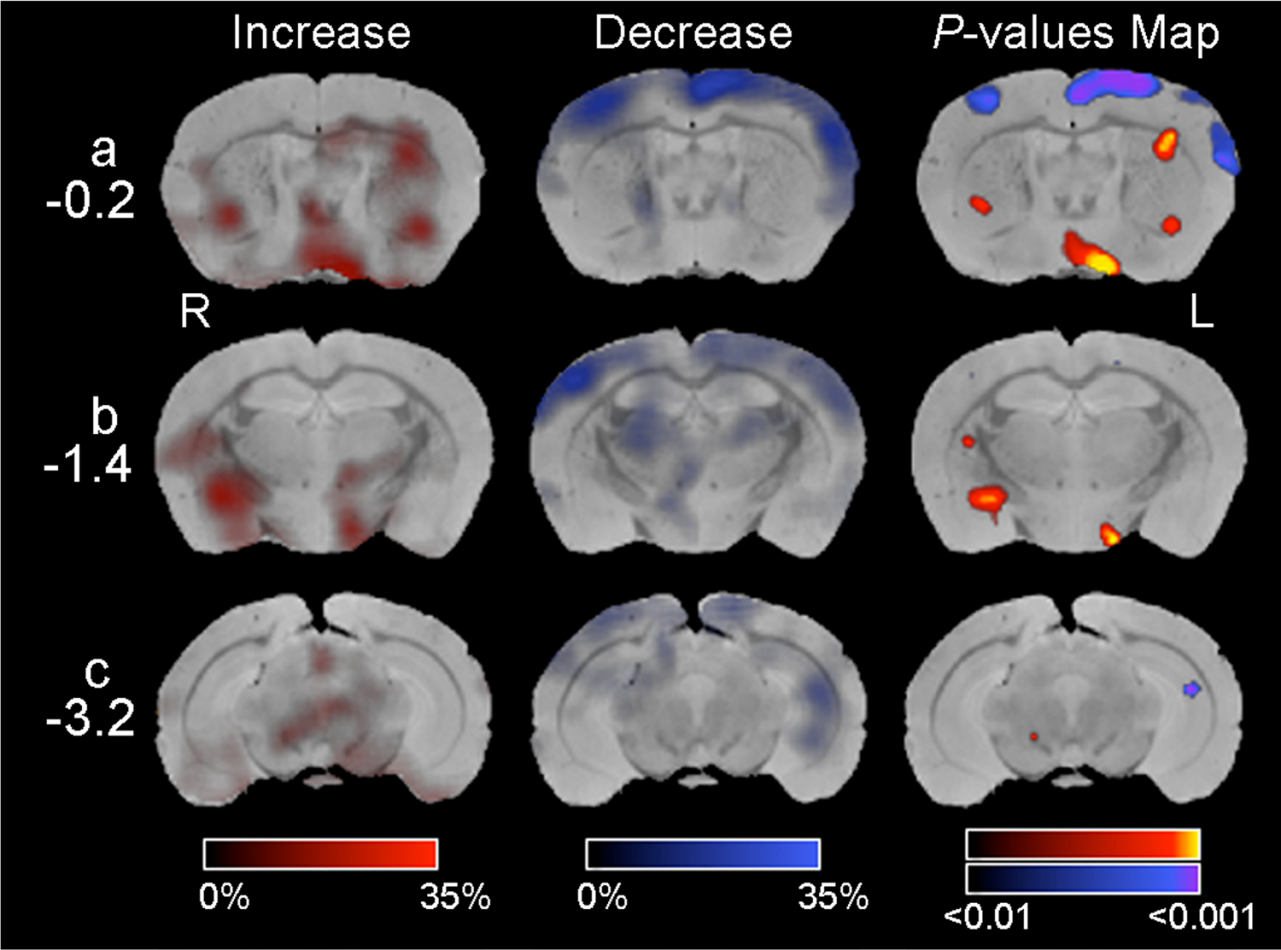
Changes of regional cerebral blood flow (rCBF) in *Nptn* ^*loxloxEmx1Cre*^ brain. Compared to control *Nptn* ^*lox/lox*^ mice, brain areas with increased rCBF in *Nptn* ^*loxloxEmx1Cre*^ mice are indicated in red (Increase, left column) and with decreased rCBF in blue (Decrease, middle column). rCBF in mutant mice was scored as % of change relative to control mice. P-values Map (right column) scores changes in rCBF according to their statistical significance level in the color-ranged bars from P < 0.01 to P < 0.001. R, right brain hemisphere. L, left. Distances from bregma are indicated (−0.2, −1.4, and −3.2).

The observed behavioral alterations may result from abnormal activities in the respective *Nptn* ^*lox/loxEmx1Cre*^ brain structures. Therefore, we analyzed the patterns of regional cerebral blood flow (rCBF) in awake mice as reported by functional^99m^TcHMPAO-SPECT-imaging^10, 43^ (Fig. 3). Although whole-brain topographies were performed, we identified specific differences with decreased 99mTc-contents in layers I-III/ IV in both left and right hemispheres of the frontal and middle sensorimotor cortex and in the ventral part of the left hippocampal CA1 region of *Nptn* ^*lox/loxEmx1Cre*^ compared to *Nptn* ^*lox/lox*^ control mice. Interestingly, the largest and highly significant decreases in ^99m^Tc-content occurred in the left somatosensory and motor cortex I and II which are contrasted by increased^99m^Tc-content in anterior and lateral hypothalamic and striatal regions in *Nptn* ^*loxloxEmx1Cre*^ mice. Notably, the highest increase of 99mTc-content in the striatum occurred within its lateral sensorimotor domain a known target of glutamatergic projection neurons from the sensorimotor cortex^44^. Cerebellar activity was not different between *Nptn* ^*lox/loxEmx1Cre*^ and *Nptn* ^*lox/lox*^ control mice (data not shown).

**Figure 4:**
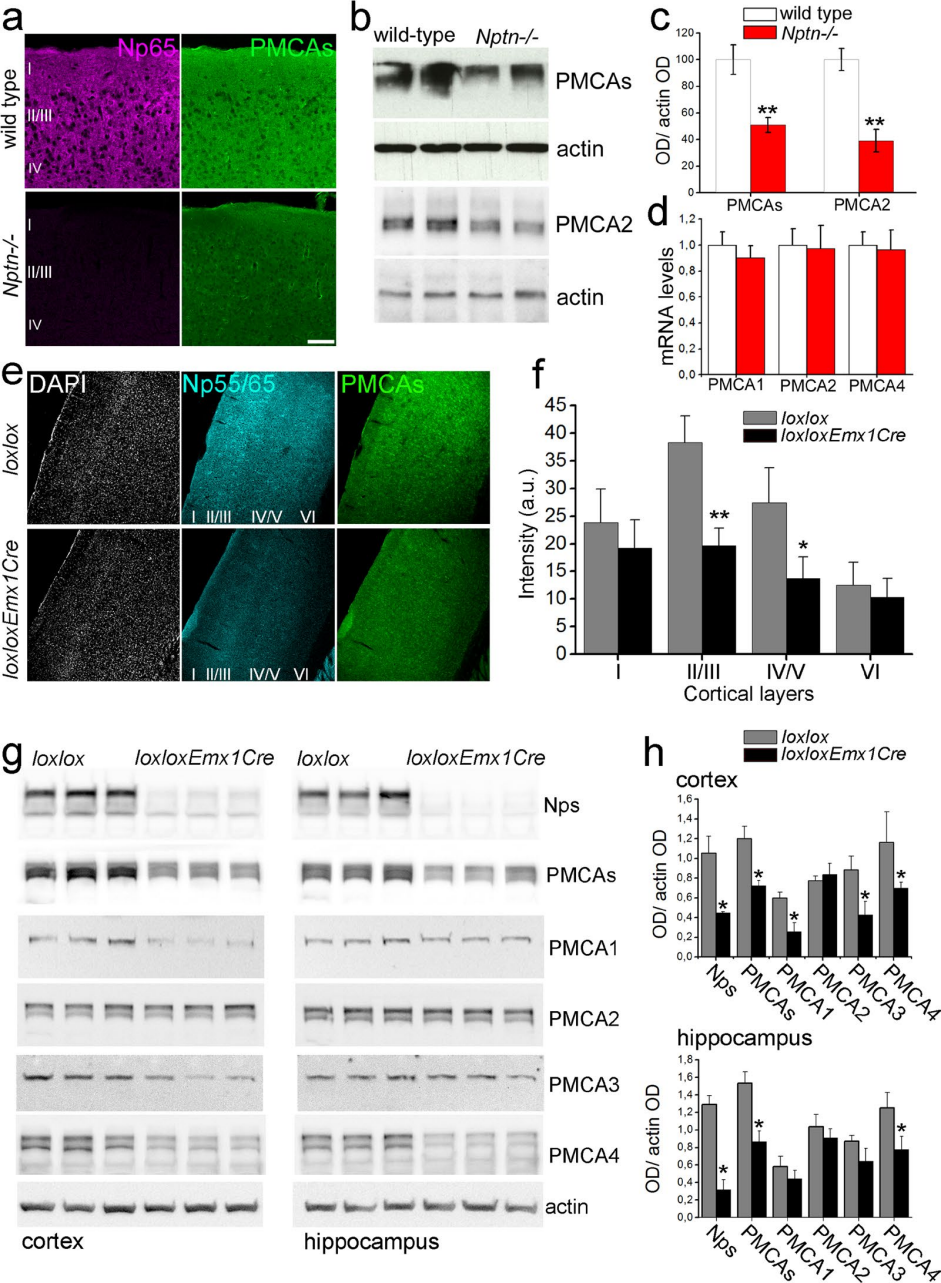
Distinct loss of PMCA isoforms in *Nptn* ^*loxloxEmx1Cre*^ brain. Reduction in total PMCA immunoreactivity was assessed using (**a**) Np65 and pan-PMCAs antibodies and confocal microscopy in *Nptn* ^−/−^ cortical slices (scale bar = 100 µm) or (**b**) pan-PMCAs and PMCA2 antibodies and Western blot analysis of homogenates from *Nptn* ^−/−^ brains. (**c**) Intensity quantification of the Western blots in **b**. Six wild-type (white bars) and six *Nptn* ^−/−^ (red bars) independent samples were quantified. (**d**) Quantitative RT-PCR for PMCA1, 2, and 4 in *Nptn* ^−/−^ brain samples. PMCA products were normalized to control GAPDH products and then, each individual mutant was normalized to the corresponding wild-type paralog value. (**e**) Representative confocal sections from *Nptn* ^*loxloxEmx1Cre*^ and *Nptn* ^*loxlox*^ somatosensory cortex stained with a pan-Np55/65 (cyan) and a pan-PMCA antibody (green) with DAPI as counterstain (grey). (**f**) Quantification of PMCA staining intensity throughout cortical layers of *Nptn* ^*loxlox*^ (gray bars) and *Nptn* ^*loxloxEmx1Cre*^ (black bars) mice (three confocal sections per animal, 4 animals per genotype were quantified as described)^10, 13^. (**g**) Homogenates from cortex and hippocampus of *Nptn* ^*loxloxEmx1Cre*^ (*loxloxEmx1Cre*) and *Nptn* ^*loxlox*^ (*loxlox*) mice were analyzed by Western blot using paralog-specific PMCA and pan-Np55/65 antibodies as indicated. Actin was used as loading control. (**h**) Intensity quantification of the Western blots in **g**. Three *Nptn* ^*loxlox*^ (gray bars) and three *Nptn* ^*loxloxEmx1Cre*^ (black bars) independent samples were quantified. Data are mean ± SEM with *P < 0.05 or **P < 0.01, unpaired two-tailed t-test. For confocal images, cortical layers are indicated.

Previously, we observed that Np-deficiency strongly reduced total PMCA expression in brain extracts and was associated with altered brain activity^10^ linking alterations in neuronal activity to [iCa^2+^] and Ca^2+^-signaling in Np animals. We further characterized PMCA expression levels by immunofluorescence staining and confocal microscopy and observed homogeneously reduced PMCA immunoreactivity in hippocampus (not shown) and cortex of *Nptn* ^−/−^ mice (Fig. 4a). Indeed, fluorescence intensity measurements revealed a reduction from 100 ± 15.8% in wild-type to 42.2 ± 19.1% in *Nptn* ^−/−^ slices (P < 0.05, six animals per genotype, unpaired two-tailed t-test). Furthermore, using an additional paralog-specific PMCA2 antibody and Western blot analysis, decreased protein levels of this neuron-specific pump in *Nptn* ^−/−^ brain homogenates were confirmed (Fig. 4b,c). Importantly, PMCA1, 2, and 4 mRNA transcript levels remained unchanged (Fig. 4d) indicating normal transcription of PMCA paralogous genes in the complete absence of Np in *Nptn* ^−/−^ mice.

Guided by the SPECT-imaging results (Fig. 3), we analyzed Np and PMCA immunoreactivities throughout the sensorimotor cortex of *Nptn* ^*lox/loxEmx1Cre*^ mice using confocal microscopy (Fig. 4e). PMCA immunoreactivity decreased in all cortical layers with the largest and significant decrease in layers II/III and IV/V (Fig. 4f, P < 0.05, four animals per genotype, three sections each, unpaired two-tailed t-test), whereas in interneurons of *Nptn* ^*lox/loxEmx1Cre*^ mice Np and PMCA immunoreactivities were preserved (Supplementary Figs S4 and S6) as expected. Protein levels of each PMCA paralog were assessed in homogenates of *Nptn* ^*lox/loxEmx1Cre*^ cortex and hippocampus using paralog-specific antibodies and Western blotting (Fig. 4g). Densitometric analysis confirmed severely reduced Np55/65 and pan-PMCA immunoreactivities in *Nptn* ^*lox/loxEmx1Cre*^ cortical and hippocampal homogenates. Compared to control *Nptn* ^*lox/lox*^, PMCA1, 3, and 4 were clearly reduced in cortex of *Nptn* ^*lox/loxEmx1Cre*^ mice (Fig. 4g,h). In the *Nptn* ^*lox/loxEmx1Cre*^ hippocampus, PMCA1, 2, and 3 were only slightly reduced, while PMCA4 was strongly reduced (Fig. 4g,h). Additionally, distinct reduction of PMCA1 immunoreactivity in cortex and hippocampus was confirmed by confocal microscopy (Supplementary Fig. S6b).

**Figure 5:**
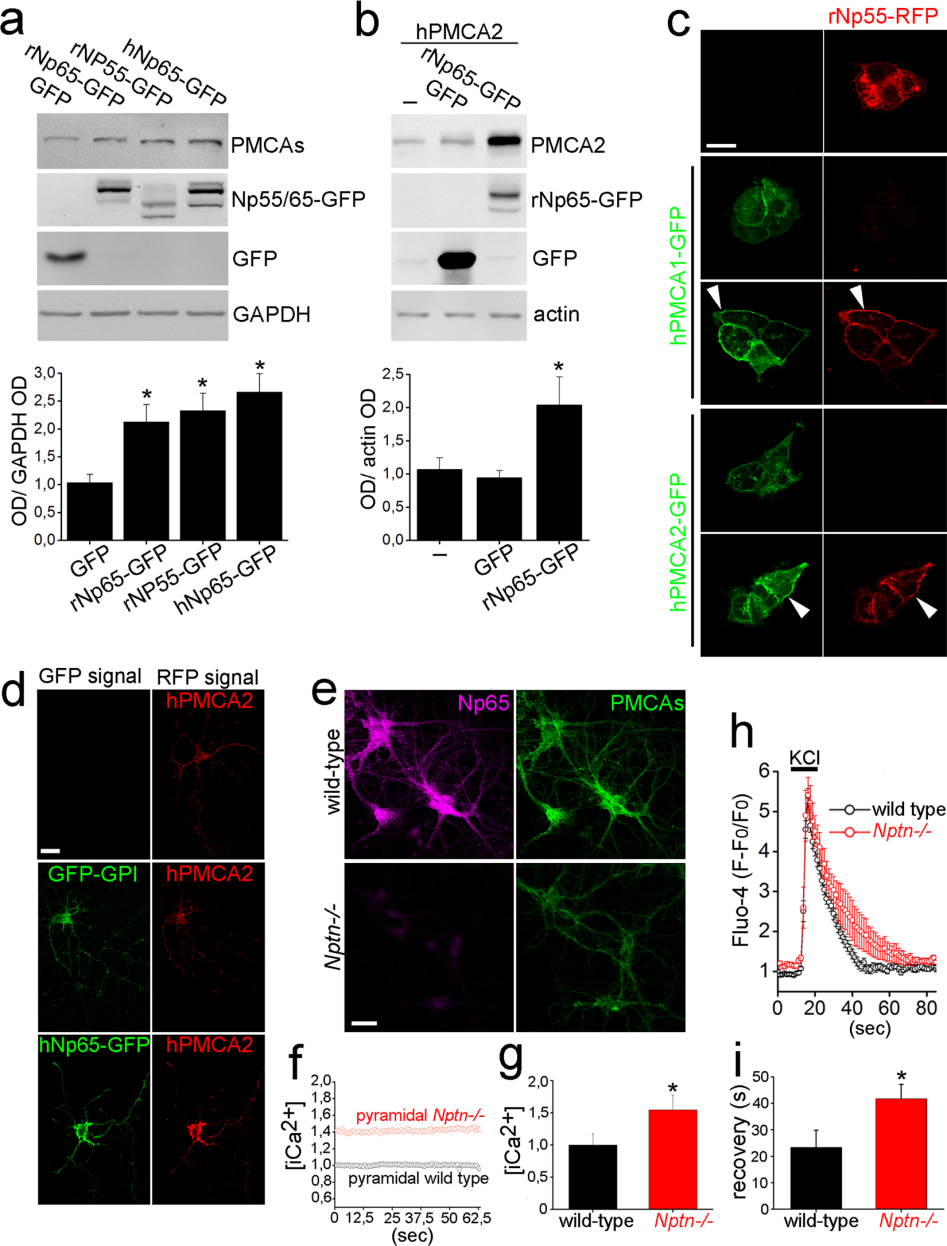
PMCA expression and [iCa^2+^] depend on Np. (**a**,**b**) Total homogenates from HEK cells were analyzed by Western blot. Identity of proteins is indicated on the right. Antibodies against GFP and GAPDH/actin were used to control protein over-expression and equal loading of the samples, respectively. Graphs displayed below each representative experiment show blot densitometries expressed as mean ± SEM (four independent experiments per series. *P < 0.05 vs. GFP, unpaired two-tailed t-test). (**a**) Endogenous PMCA levels in cells transfected 24 hours with GFP, rat Np65-GFP (rNp65-GFP), rNp55-GFP, or human Np65-GFP (hNp65-GFP) were assessed using a pan-PMCA antibody. (**b**) HEK cells were transfected with hPMCA2 or co-transfected with GFP or rNp65-GFP additionally. hPMCA2 was detected with an anti-PMCA2 antibody. (**c**) Single or double transfection in HEK cells with plasmids encoding rNp55-RFP and hPMCA1-GFP or hPMCA2-GFP. Arrow heads point to PMCA1-Np55 or PMCA2-Np55 co-localization in the cell plasma membrane of co-transfected cells. Representative pictures from 3 independent transfections. (**d**) Immature neurons (5 DIV) were transfected with hPMCA2 (red) or co-transfected with hPMCA2 and GFP-GPI or hPMCA2 and hNp65-GFP for 24 hours and photographed using confocal microscopy. hPMCA2 over-expression was revealed using an anti-PMCA2 antibody and a Cy3-congugated secondary antibody. Representative pictures from 3 independent transfections. (**e**) Mature wild-type and *Nptn*−/− neurons (16 DIV) were stained with antibodies against Np65 (magenta) and pan-PMCAs (green) and photographed using a confocal microscope (representative pictures from at least 5 independent experiments) or (**f**,**g**) loaded with Fluo-4 and Fura-Red fluorescent probes to measure [iCa^2+^]. (**f**) *Nptn*−/− intensity ratio was normalized against wild-type ratio and demonstrates the stability of the single-cell recordings in the presence of 1 µM TTX. (**g**) The graph shows the baseline intensity ratio expressed as mean ± SEM (16 wild-type and 18 *Nptn*−/− neurons (*P < 0.05, unpaired two-tailed t-test). (**h**, **i**) Fluo-4-loaded mature wild-type and *Nptn*−/− neurons (16 DIV) were stimulated with KCl (30 mM, 15 sec). (**h**) The spikes are the mean ± SEM of 11 wild-type and 12 *Nptn*−/− neurons. (**i**) Time required to recover to baseline levels of [iCa^2+^] (peak-to-baseline) was quantified (*P < 0.05, unpaired two-tailed t-test). (**f**,**g**,**h**,**i**) Measurements were performed in pyramidal neurons as identified morphologically in a confocal microscope under controlled conditions of temperature and pH. Upper graph Lower Scale bars = 20 µm.

To gain insight into the molecular mechanisms underlying the Np-PMCA association, we tested in cultured cells the hypothesis that Np modulates PMCA levels directly. In HEK cells, a human cell line not expressing Nps nor PMCA2^40, 45^, all three GFP-tagged versions of Nps, namely rNp55, rNp65, and hNp65 increased endogenous hPMCA protein levels as evaluated by Western blot in total extracts (Fig. 5a upper panel) and confirmed by quantification of the optical density of the bands (Fig. 5a lower graph). rNp65-GFP was effective to increase consistently the basal over-expression of exogenous hPMCA2 (Fig. 5b) in transiently transfected HEK cells. Furthermore, the expression and plasma membrane localization of exogenous hPMCA1-GFP and hPMCA2-GFP were promoted robustly by rNp55-RFP as evaluated by confocal microscopy (Fig. 5c). Immature rat hippocampal neurons cultured for 5 days express low levels of endogenous Np65 and PMCA2^23, 46^. As in HEK cells, hNp65-GFP cDNA constructs promoted the expression of exogenous hPMCA2 (Fig. 5d) in co-transfected immature neurons with large and pyramidal soma. Therefore, both rat and human Np can promote PMCA protein levels in both human and rat cultured cells.

Because PMCA is the only high affinity extrusion mechanism able to fully restore basal [iCa^2+^], reduced levels or compromised activity of PMCA result in elevated basal [iCa^2+^] and/or reduced clearance of the ion^21, 22, 34, 47^. In agreement with our data showing that PMCA expression in glutamatergic neurons depends on Np *in vivo*, mature pyramidal Np-deficient neurons cultured for 16 days displayed reduced PMCA levels in comparison to wild-type (Fig. 5e). As expected, we obtained stable measurements of [iCa^2+^] using single-cell Fluo-4/Fura-Red ratiometric Ca^2+^ and confocal imaging in the absence of any potentially interfering intrinsic neuronal network activity (1 µM TTX) (Fig. 5f). [iCa^2+^] was elevated by 51.9 ± 2.1% in pyramidal glutamatergic neurons lacking Np compared to wild-type neurons (Fig. 5g, *P* < 0.05, unpaired two-tailed t-test). Elevated basal [iCa2+] was also observed in Fluo-4-loaded mutant neurons after fluorescence intensity normalization to wild-type neurons (Fig. 5h). Consistent with the literature^34^, basal [iCa2+] was restored after washed-out of KCl (30 mM, 15 sec) with a recovery delay of 23.4 ± 6.4 sec in wild-type neurons (Fig. 5h,i). In contrast, slow (Fig. 5i, P < 0.01, unpaired two-tailed t-test) and inefficient restoring of basal [iCa2+] occurred in Np-deficient neurons further indicating insufficient PMCA activity after KCl stimulation. Differences in rise time or maximal response were not observed between the phenotypes (Fig. 5h).

## Discussion

Here, we show that Np ablation in cortical and hippocampal glutamatergic neurons – the predominant neuron type expressing Np in human and rodent brain – results in strong and specific behavioral deficits correlating well with decreased neural activities and reduction of PMCAs in the mutant brain areas. Although Nps are capable of regulating directly the protein levels of PMCAs in cultured cells *in vitro*, the Np-dependent post-transcriptional expression of each specific PMCA differs between neuron-types in cortex and hippocampus *in vivo*. These results provide new molecular grounds to link the causality of Np ablation to alterations in [iCa^2+^] and neuronal activities in cognitive circuits. As the expression, molecular features, and synaptic localization of Np in human and rodent hippocampus and cortex are very similar, the revealed mechanisms of learning and memory are translatable to conditions underlying cognition and its deterioration.

The behavioral analysis of *Nptn* ^*lox/loxEmx1Cre*^ mice allowed us to identify functions mediated by Np expressed in glutamatergic neurons, which appear to be molecularly distinct from other Np functions e.g. in GABAergic neurons^13, 48^. Using the water maze, we observed that *Nptn* ^*lox/loxEmx1Cre*^ mice formed a reliable spatial memory, indicated by their memory for the goal quadrant. However, *Nptn* ^*lox/loxEmx1Cre*^ mice were not handicapped by moving the platform position during reversal but displayed a more circular alternative strategy indicative for a shift from use of hippocampus-dependent distal to striatum-dependent proximal cues^42^. In Np65-deficent mice, a role of Np in hippocampus-dependent use of spatial information has been suggested^17^ and is supported functionally by insufficient mEPSC and synaptic plasticity in the CA1 region^10^ as well as anatomically by the reduced number of excitatory synapses^13^. Additionally, the prolonged immobility and frequent re-initiation of swimming by *Nptn* ^*lox/loxEmx1Cre*^ mice in the water maze caused a delay to reach the platform during acquisition and suggests further alterations in the cortical-striatal pathways related to decision processing^49, 50^.

In the two-way active avoidance (shuttle-box) paradigm, *Nptn* ^*lox/loxEmx1Cre*^ mice acquired the associative fear conditioning task normally contrasting strongly with *Nptn* ^−/−^ mice and *Nptn* ^*lox/loxPrpCreERT*^ induced when adult which both do not learn this task^10^. Strong evidence indicates that disinhibition –“a transient break in the balance of excitation and inhibition”- of glutamatergic projection neurons mastered by GABAergic interneurons is crucial for acquisition and expression of associative fear conditioning^5^. In *Nptn* ^−/−^ and induced *Nptn* ^*lox/loxPrpCreERT*^ mice, Np is lacking in GABAergic neurons causing altered GABAergic transmission^10, 13^ and potentially affecting disinhibition resulting in inability to acquire this associative task. In contrast, GABAergic interneurons express Np and may be able to regulate inhibition in the glutamatergic *Nptn* ^*lox/loxEmx1Cre*^ mutant. In favor of this assumption, our results show that expression of Np in glutamatergic neurons is not essential for the acquisition. Strikingly, we identified unequivocally abundantly Np expressing parvalbumin-containing GABAergic interneurons within the cortical layer II/III (Supplementary Results), which are known as a fundamental neuron type mediating disinhibition^5^. The hypothesis of Np participating in GABAergic disinhibition of glutamatergic neurons fits to previous reports relating Np with the balance of the ratio of excitatory/inhibitory synapses^10, 13, 17^ *via* fundamentally independent synaptic mechanisms^13^.

The capacity of *Nptn* ^*lox/loxEmx1Cre*^ mice to form associative memories argues against a role of Np expressed by glutamatergic neurons in this process. In contrast, in trained *Nptn* ^*lox/loxPrpCreERT*^ mice loss of Np in all neuron types elicits retrograde amnesia of associative memories^10^ and suggests the requirement of Np expressed by other neurons for the retrieval of memories.

In resting *Nptn* ^*lox/loxEmx1Cre*^ mice, we observed altered patterns of ^99m^Tc-content in those key neuronal circuits known to be directly involved in the processing of the analyzed behaviors. As disturbances in neuronal circuit activities are reflected in detectable changes of ^99m^Tc-content^10, 43^, we interpret that decreased activities in the mutant hippocampal CA1 and sensorimotor cortex layers II/III are immediate consequences of Np ablation in glutamatergic neurons. Increased activity in the sensorimotor domain of the striatum might result from cortex-striatum circuit adaptation to decreased activity of the mutant sensorimotor glutamatergic afferents^44^. This striking correlation of permanently altered resting brain activity with compromised specific behavioral performance indicates that the loss of Np in glutamatergic neurons affects basal properties of these neuronal circuits.

The characterization of PMCA isoforms, whose expression is depending decisively on Np, establishes a mechanistic ground linking causally Np ablation to alterations of activities and functions of the mutant cortical and hippocampal areas. PMCA reduction cannot be explained as an adaptive compensatory phenomenon during development in *Nptn* ^−/−^ or *Nptn* ^*lox/loxEmx1Cre*^ mice as it can be induced efficiently and similarly in adult *Nptn* ^*lox/loxPrpCreERT*^ mice^10^. Also, a drastic reduction on PMCA1, 3, and 4 levels occurred in the cortical layers II/III in *Nptn* ^*lox/loxEmx1Cre*^ mice - coincidently the layers with the highest expression of Np and PMCA in wild-type cortex - arguing for a direct dependency of these paralogs on Np expression in cortical glutamatergic neurons. Very interestingly, PMCA2 levels remained grossly unchanged in *Nptn* ^*lox/loxEmx1Cre*^ brain but were clearly reduced in *Nptn* ^−/−^ brain. Thus, neuron types other than glutamatergic neurons may express most of the PMCA2 sensitive to Np ablation. Parvalbumin-positive interneurons are good candidates to play this role as we identified them expressing Np and PMCA abundantly and they are known to express particular PMCA2 splicing variants^27^. Considering PMCAs as key regulators of neuronal activity and synaptic plasticity^45, 51, 52^, it is very likely that the reduction of cortical PMCA1, 3, and 4 contributed directly to decreased cortical activity in *Nptn* ^*lox/loxEmx1Cre*^ mice. Similarly, loss of hippocampal PMCA4 may be related to decreased activity in the mutant CA1 hippocampal area. Although hippocampal PMCA1, 2, and 3 were only slightly reduced it is not possible to fully exclude that the collective decrease of these three paralogs could further contribute to [iCa^2+^] alterations in hippocampal circuit activity.

Reduction of PMCA protein was not accompanied by changes of mRNA paralog levels *in vivo*. Using cDNA transfection of human or rat Np55 or Np65 in human or rat cultured cells, we demonstrated that either exogenous Np55 or Np65 is sufficient to promote PMCA expression independently of the cellular transcriptional program. Thus, we conclude that Nps have the capacity to modulate PMCA levels post-transcriptionally. Indeed, human and rat Np55/65 increased levels of and co-localized in the cell plasma membrane with hPMCAs suggesting the conserved function between species. Furthermore, our recent findings indicate that PMCAs encoded by all four paralogs form complexes with Np55/65 with a defined stoichiometry (Dr. Thilo Kähne, unpublished data) in the neuronal plasma membrane to maintain low basal [iCa^2+^]. It is tempting to speculate, that Np may stabilize PMCA or withdraw it from degradation by forming complexes. In the light of our data confirming Np as necessary for diverse and/or specialized PMCA expression *in vivo* for example PMCA1, 3 in cortical glutamatergic neurons, we propose that specific Np-PMCA complexes regulate differentially [iCa^2+^]-homeostasis and neuronal activity of particular neuron types in brain areas related to cognitive processing.

Recently a genome-wide association (GWA) study of intelligence differences on 1,500 adolescents concluded that Np gene expression accounted for 0.5% of the variance of cognitive capacities^15^. Although, GWA studies have contributed to the understanding that many genes exert small effects on the heritability of intelligence, the association of Np with intelligence has been diminished based on a larger analysis of 18,000 subjects^38^. Therefore, the exact relation of Np expression and high cognitive functions as well as the contribution to this trait in a given genetic background or social environment remain to be determined. Evident similarities in expression and molecular features between human and rodent Np further support the translational value of Np animal models for learning and memory. We described prominent inter-species similarities in Np distribution in the principal excitatory pathways across the glutamatergic circuits within human hippocampus and entorhinal cortex, namely strong hNp expression in the cell-containing layers, in the somatic and neuropil layers of the CA1, in the inner molecular layer of the DG and in pyramidal neurons from the layer II and IV/V in the entorhinal cortex. Despite the highly specific and efficient hNp immunodetection, we did not observe relative enrichment of hNp immunoreactivity in the CA1 area compared to CA3 or DG contrasting with the rodent CA1 region. This difference can be explained by anatomical variations in position in the used human sections as well as known histological differences such as larger CA1 and CA2 areas and thinner DG polymorphic layer in humans vs. rodents^53, 54^. As in rodents, hNp mRNA levels increase rapidly after birth, are high during adolescence-young adulthood, and decline with age. After translation, hNp mRNAs yield the two main glycosylated isoforms hNp55 and hNp65, which co-exist in close proximity in the neuronal plasma membrane of glutamatergic neurons and in glutamatergic synapses. Interestingly, Np55 and Np65 are abundant in human PSDs contrasting rodent PSDs where only Np65 is detected suggesting a greater demand of Np in human glutamatergic transmission, for example, to regulate PMCA levels. Deregulated expressions of PMCA4^31^ and Np^16^ have been reported independently in schizophrenic patients. Interestingly, the severely impaired startle response and prepulse inhibition revealed a strong schizophrenic component in the behavior of *Nptn* ^−/−^ and *Nptn* ^+*/*-^ mice^10^. Very recently, autism has been associated to low expression of ATP2B2^29, 30^, the human gene encoding PMCA2, which may be compared with decreased PMCA2 in *Nptn* ^−/−^ mice displaying altered social behavior^10^.

The experimental approach using Np ablation in mice is a promising unique tool for studying mechanisms related to learning and memory and cognitive dysfunctions. As we show here, brain area- and neuron type-selective Np ablation may not only allow the delineation of causal molecular mechanisms in specific neuronal circuits influencing higher brain processes, but may also model important aspects of iCa^2+^-homeostasis dysregulation linked to cognitive deterioration and potentially to neuropathological conditions.

## Methods

### Human mRNA expression and brain material

All procedures involving human subjects were done in accordance with the approval of the Central Ethical Committee of the University of Zagreb Medical School case no. 380-59/11-500-77/90, class 641-01/11-02 signed on 19th May 2011. We used http://BrainCloud.jhmi.edu ^55^ to evaluate gene expression (mRNA levels) in dorsolateral prefrontal cortex from 215 subjects (0 to 80 years old) with RNA integrity number ≥8 as reported^15^. Probes targeting translated mRNA regions were selected. Human material from six subjects with no history of neuropsychiatric diseases and cause of death unrelated to neurological disorders or head trauma (mean age: 69.5; range: 59–81 years old) was obtained from the Zagreb brain collection^56^ and the Brain Bank at Karolinska Institute (Stockholm, Sweden) with permission of the ethical committees. For staining, brains were fixed (10% pH neutral formalin for 21 days) and hippocampal-entorhinal cortex was cut in rostro-caudal direction. We used five 12 μm thick sections for Nissl staining to delineate cytoarchitectonic boundaries. Frozen brain samples derived from one additional subject were processed for biochemical analysis, as described below.

### DAB staining

After dewaxing and rehydration, human brain sections were incubated in citrate buffer (pH 6.0, 20 min, 95 °C), treated with 0.3:25:74.7 hydrogen peroxide:methanol:water (20 min), immersed in blocking solution (5% horse serum, 0.5% Triton X-100 in PBS, Sigma) for two hours, and incubated with goat anti-Np65 in blocking solution (1:500, R&D Systems, overnight, 4 °C). Incubation with anti-goat secondary antibody conjugated with horse-radish peroxidase (1:1000, Jackson Immunolaboratories) in blocking solution followed (one hour, room temperature). Negative controls included pre-immune serum replacing primary antibody, omission of secondary antibody, or use of non-related secondary antibody. Sections were visualized using diaminobenzidine (DAB) and scanned (453 nm/pixel) using a Hamamatsu NanoZoomer C10730-12. Signal intensities were scored using a bright-field microscope by three independent researchers as none (0), low (+), moderate (++), or strong (+++). Additional quantification of immunoreactivity used ImageJ densitometric analysis.

### Neuroplastin mutant mice, behavior, and SPECT-imaging

*Nptn* ^−/−^ and floxed *Nptn* ^*loxlox*^ mice were described^10, 13^. To obtain *Nptn* ^*loxloxEmx1Cre*^ mice, B6.129S2-Emx1^*tm1*(*Cre*)^ (The Jackson Laboratory) were crossed with *Nptn* ^*loxlox*^ mice and maintained on a *Nptn* ^*loxlox*^ background. Mice were kept with a 12-hour light/dark cycle and food and water *ad libitum*. All procedures were in accordance with institutional, state, and government regulations (Landesverwaltungsamt Sachsen-Anhalt, permit 42502-2-1366 LIN). Associative learning was assessed by two-way active avoidance in a shuttle-box, spatial learning in the hidden platform Morris Water Maze, and motor coordination using a rota-rod as described^10, 57^ and detailed in Supplementary Material and Methods. Small animal single photon emission computed tomography (SPECT) imaging of regional cerebral blood flow (rCBF) was performed *in vivo* mapping spatial patterns of neuronal activity as published^10, 43^ and described in Supplementary Material and Methods.

### Subcellular fractionation, protein deglycosylation, Western blot analysis, and Immunofluorescence

Samples were stored at −80 °C until fractionation experiments, unfrozen in ice-cold buffer (320 mM sucrose, 5 mM HEPES, pH 7.4), and homogenized. Subcellular fractions were obtained as described^58^. Deglycosylation, Western blot analysis, and Immunofluorescence are described in Supplementary Material and Methods.

### Cell cultures, cDNA transfections, and iCa^2+^ measurements

Np mutant hippocampal neurons were prepared and cultured for 12–16 DIV as described^13^. Human Embryonic Kidney (HEK) cells were maintained as recommended by ATCC. Cells were transfected using Lipofectamine 2000 as recommended and analyzed after 24 hours. cDNAs: Human PMCA2 constructs (ID: 47581 and 47584) and PMCA1 (ID:47758) were purchased from Addgene (Cambridge, MA), rat Np constructs were described^40^, and human Np65 (OHu06230D, Genscript, Piscataway, NJ) was inserted into pFUGW-EGFP (Addgene ID:14883). For single cell ratiometric iCa^2+^ imaging, cell somata were loaded with Fluo-4 (1.3 μg/ml) and Fura-Red (2.7 μg/ml) fluorescent probes for 30 minutes. A single wavelength laser (488 nm) was used for stimulation, and fluorescence emission was collected dually at 520 and 680 nm. Ratio of fluorescent intensities was normalized against the basal ratio of wild-type neurons as required. Measurements were performed with a confocal microscope under controlled conditions of temperature and pH. To prevent interference of spontaneous action potentials, intrinsic neuronal activity was silenced using 1 μM TTX in the recording bath (145 mM NaCl, 2.5 mM KCl, 2 mM MgCl_2_, 2 mM CaCl_2_, 10 mM HEPES, and 10 mM D-glucose, pH 7.4). Perfusion (2 ml/min) of isosmotic 30 mM KCl for 15 sec was performed according published protocols^34^ and using a RC-37R chamber from Warner Instruments (Hamden, CT, USA).

### Statistical analysis

Statview (SAS Institute Inc., Cary, NC) was used for Analysis of Variance (ANOVA), post hoc analysis (Scheffe’s or Fisher PLSD), and repeated measures ANOVA. In the voxelwise analysis unpaired t-tests were made to compare brain ^99m^Tc distributions using the MagnAn-software (version 2.4, BioCom, Germany) and uncorrected p-values^59–61^.

### Data Availability

The datasets generated during and/or analyzed during the current study are available from the corresponding author on reasonable request.

## Electronic supplementary material

Supplementary Information
