# Supplementary material for: Neuroplastin deletion in glutamatergic neurons impairs selective brain functions and calcium regulation: implication for cognitive deterioration
Source: Sci Rep. 2017 Aug 4;7:7273. doi: 10.1038/s41598-017-07839-9 (PMC5544750; doi:10.1038/s41598-017-07839-9)
Supplement: Supplementary file 1 — Supplementary Information [file 41598_2017_7839_MOESM1_ESM.pdf]

**Supplement**

# Neuroplastin deletion in glutamatergic neurons impairs selective brain functions and calcium regulation: implication for cognitive deterioration

**Short title**: Neuroplastin deletion impairs brain functions

Rodrigo Herrera-Molina Ph.D.[1*], Kristina Mlinac-Jerkovic Ph.D.[2*], Katarina Ilic M.D.[2*], Franziska Stöber M.SC.[3], Sampath Kumar Vemula[1], Mauricio Sandoval Ph.D.[1], Natasa Jovanov Milosevic Ph.D.[2], Goran Simic Ph.D.[2], Karl-Heinz Smalla Ph.D.[4], Jürgen Goldschmidt M.D.[3], Svjetlana Kalanj Bognar PhD[2], Dirk Montag Ph.D[5#]

*these authors contributed equally

Departments of [1]Neurochemistry and Molecular Biology and [3]Systems Physiology; Special Laboratories for [4]Molecular Biology Techniques and [5]Neurogenetics; Leibniz Institute for Neurobiology, Magdeburg, Germany

[2]Croatian Institute for Brain Research, School of Medicine, University of Zagreb, Zagreb, Croatia

[#] for correspondence:

Dirk Montag, Neurogenetics Special Laboratory, Leibniz Institute for Neurobiology, Brenneckestrasse 6, 39118 Magdeburg, Germany; telephone number: +49-391-6263-94241; email address: montag@lin-magdeburg.de

**Supplementary Material and Methods**

**Behavior**

Briefly, associative learning was assessed by two-way active avoidance in a two-chambered shuttle-box (TSE, Bad Homburg, Germany) with 10 sec light as conditioning (CS) and electrical foot-shock as unconditioned stimulus (US, 5 sec, 0.5 mA pulsed) delivered after the CS (80 trials/ day, 5-15 sec stochastically varied inter-trial intervals, 5 consecutive days)[1]. Compartment changes during CS were counted as conditioned avoidance reactions. Motor capabilities were assessed by the latency to fall off the rota-rod (TSE) and were determined in two training sessions (3 hrs interval) with increasing speed (4 to 40 rpm, 5 min) and 4 days later at 16, 24, 32, and 40 rpm constant speed[1]. Spatial learning was assessed in the hidden platform Morris Water Maze using VideoMot2 (TSE) and Wintrack software[1,2]. Grip strength was measured with a force sensor (TSE Systems GmbH, Bad Homburg, Germany)[1].

**SPECT-imaging**

*Nptn*$^{loxloxEmx1Cre}$ mice (n = 9) and controls *Nptn*$^{loxlox}$ mice (n = 10) were injected *via* jugular vein catheters with 130.61 ± 6.32 MBq of $^{99m}$Tc hexamethylpropyleneamine oxime ($^{99m}$Tc-HMPAO) with a flow rate of 26-35 µl/min for 15 min. After $^{99m}$Tc-HMPAO-injection animals were anesthetized and scanned using a four head NanoSPECT/CT scanner (Mediso, Hungary). CT and SPECT images were co-registered. CT scans were made at 45 kVp, 177 µA, with 180 projections, 500 msec per projections, and reconstructed with the manufacturer's software (InVivoScope 1.43) at isotropic voxel-sizes of 100 µm. 24 projections were acquired during a total scan time of 1 hour. Axial FOV was 20.9 mm. Energy windows were set to the default values of the NanoSPECT/CT (140 keV ± 5%). SPECT images were reconstructed using the iterative algorithm of the manufacturer's software (HiSPECT, SCIVIS, Goettingen, Germany) at isotropic voxel output sizes of 250 µm. For data analysis brain $^{99m}$Tc-distributions were compared in *Nptn*$^{loxloxEmx1Cre}$ mice and *Nptn*$^{loxlox}$ controls. SPECT/CT images were manually aligned to a high-resolution MR mouse brain data set[2,4] based on skull-landmarks of the CTs with the MPI-Tool software (version 6.36,

Advanced Tomo Vision, Kerpen, Germany). SPECT brain data were cut out of the SPECT data in Osirix (64-bit, version 5.7.1; Pixmeo, SARL, Bernex, Switzerland) using a whole-brain volume-of-interest (VOI)[3,4]. Brain SPECT data were global mean normalized using the MPI-Tool software.

### Subcellular fractionation and Western blot analysis

After centrifugation of homogenates at 1000 **g**, the supernatant was centrifuged at 12000 **g** for 20 min. The resulting pellet (P2, total membrane fraction) was fractionated on a sucrose step gradient to obtain the synaptosomal fraction (1.0/1.2 M sucrose interface) which was then lysed in 5 vols of a hypo-osmotic solution (5 mM Tris/HCl, pH 8.1) for 30 min and centrifuged at 33000 **g** for 30 min. The resulting pellet was resuspended for a second sucrose gradient to yield synaptic junctions. This fraction was suspended in 320 mM sucrose/10 mM Tris/HCl (pH 8.1) and was delipidated by mixing with an equal volume of 320 mM sucrose/1% Triton X-100 (60 ml/10 g wet tissue). The suspension was kept on ice for 15 min and centrifuged for 30 min at 33000 **g**. Postsynaptic densities were resuspended in 5 mM Tris/HCl, pH 8.1. After quantification, proteins were separated by SDS gel electrophoresis and transferred onto nitrocellulose. Membranes were incubated overnight with primary antibodies: goat anti-Np65 or sheep pan-Np55/65 (1:2000, R and D Systems); or pan-PMCA (clone 5F10) or anti-PMCA4 (clone JA9) (1:1000, Abcam); or anti-PMCA1, anti-PMCA2 or antiPMCA3 raised in rabbit (1:1000, Abcam). Then, immunoreactivity was visualized using proper peroxidase-conjugated secondary antibodies and an ECL detection system.

### Deglycosylation assay

Deglycosylation was performed using the Glyco Profile IV chemical deglycosylation kit (Sigma). Triton X-100 solubilized samples were incubated with 4 volumes ethanol at -20°C overnight, centrifuged at 15,000xg for 15 min, and pellets were washed with 80% ethanol. The pellet was transferred to reaction vials and lyophilized. Further steps were performed as

described by the manufacturer.

**Mouse hippocampal slices**

To imitate post-mortem conditions of human brains, the heads of wild-type and *Nptn*$^{-/-}$ mice were maintained for four or eight days at 4°C. Brains were removed and fixed in 4% PFA for 48 hours. After cutting, hippocampal slices were immediately attached to glass coverslips and air-dried at room temperature for 1 hour. Fluorescent staining was performed as described below for human sections. To obtain *Nptn*$^{loxloxEmx1Cre}$ brains, cardiac perfusion of 4% PFA in PBS was performed followed by further fixation with 4% PFA at 4° C for 24 hours. *Nptn*$^{loxloxEmx1Cre}$ brains were maintained in 20% (24 hours) followed by 30% sucrose (24 hours), frozen in OCT compound (Sakura Finetek), cut into 35-µm slices, and processed free-floating.

**Immunofluorescence**

Human or mouse hippocampal slices were blocked (20% horse serum in PBS, one hour, room temperature) and incubated with goat anti-Np65 (1:500) and sheep pan-Np55/65 (1:500) primary antibodies in PBS containing 0.3% Triton X-100 and 10% horse serum (overnight, 4°C). Primary antibodies were detected using Cy3-conjugated anti-sheep and Cy5-conjugated anti-goat secondary antibodies (1:1000, Jackson Immunoresearch). Sections were mounted with Mowiol. Staining of PFA-fixed *Nptn*$^{loxloxEmx1Cre}$ slices was performed as described previously[5,6]. Additional primary antibodies used for *Nptn*$^{loxloxEmx1Cre}$ slices were: pan-PMCA (clone 5F10, 1:1000), rabbit anti-PMCA1 (1:500), rabbit anti-PMCA2 (1:500), rabbit anti-parvalbumin (1:500, Thermo Scientific), and mouse anti-calretinin (1:500, Swant).

## Supplementary Results

### Np antibodies specifically stain and detect Np in mouse and human material

Specificity of pan-anti-Np55/65 and anti-Np65 antibodies was challenged by staining of wild-type and *Nptn*$^{-/-}$ hippocampal slices under similar conditions as for human samples (Supplementary Fig. S1). Evaluation using confocal microscopy confirmed that Np immunostaining persisted in wild-type whereas no signal was detected in *Nptn*$^{-/-}$ sections from mice with a post-mortal delay of 4 or 8 days. Furthermore, the anti-Np65 antibody was able to precipitate only the Np65 isoform from Triton X-100-solubilized human hippocampal extracts (Supplementary Fig. S1).

### Expression of Np in human striatum

Analysis of Np immunoreactivity in adult human basal ganglia detected specific Np labeling of neuronal cell bodies and fibres in striatum (nucleus caudatus, putamen) and globus pallidus, confirming previous observations by Bernstein et al.[7] (Supplementary Fig. S2).

### Staining of mouse and human hippocampal slices with Np antibodies

Positioning of Np55/65- and Np65-associated immunoreactivity was found substantially similar and largely co-located in intact plasma membranes of human and mouse hippocampal neurons stained with antibodies and photographed using confocal microscopy (Supplementary Fig. S3).

### Characterization of *Nptn*$^{lox/loxEmx1Cre}$ mice

*Nptn*$^{lox/loxEmx1Cre}$ mice were created by combining Emx1-directed Cre recombinase expression in glutamatergic neurons of the neocortex and hippocampus[8,9] with Cre recombinase-driven deletion of the floxed exon1 in the *Nptn* gene (Supplementary Fig. S4). Detailed examination of Np expression using confocal microscopy confirmed that Np ablation was effective in glutamatergic neurons only whereas GABAergic neurons i.e. calretinin- or parvalbumin-positive interneurons in layers II/III of the sensorimotor cortex or in the CA1 region of the

hippocampus retained their capacity to express Np in the mutant brain structures. Other brain structures such us putamen, striatum, hypothalamus retained their normal expression of Np fully (Supplementary Fig. S4).

**PMCA in interneurons and PMCA1 in *Nptn*$^{lox/loxEmx1Cre}$ mice**

As cortical and hippocampal parvalbumin-positive interneurons retained the expression of Np in *Nptn*$^{lox/loxEmx1Cre}$ mice (Supplementary Fig. S4), we combined anti-parvalbumin and anti-PMCA antibodies to evaluate the expression of PMCA in parvalbumin-positive Np-expressing interneurons. Using confocal microscopy, we observed that parvalbumin-positive interneurons are reliably identifiable as the cell type displaying the highest PMCA immunoreactivity in the glutamatergic *Nptn*$^{lox/loxEmx1Cre}$ background (Supplementary Fig. S6a). In contrast, the expression of both Np and PMCA cannot be related to single cells in the wild-type animals due to their high expression and compaction of the signals (Fig. 4, Supplementary Fig. S4 and S6a).

PMCA1 levels were strongly reduced in homogenates from *Nptn*$^{lox/loxEmx1Cre}$ cortex but only slightly in homogenates from *Nptn*$^{lox/loxEmx1Cre}$ hippocampus compared to the wild-type (Fig. 4). To confirm these results, *Nptnlox*$^{/loxEmx1Cre}$ and wild-type cortical-hippocampal slices were stained simultaneously with PMCA1 and Np65 antibodies and DAPI and photographed using confocal microscopy. Consistent with Western blot analysis in Figure 4, reduction on PMCA1 immunoreactivity was strong in *Nptn*$^{lox/loxEmx1Cre}$ cortex but moderate in *Nptn*$^{lox/loxEmx1Cre}$ hippocampus compared to wild-type (Supplementary Fig. S6b).

## Supplementary References

1. Montag-Sallaz, M. & Montag, D. Severe cognitive and motor coordination deficits in tenascin-R-deficient mice. Genes Brain Behav. 2, 20-31 (2003).

2. Wolfer, D. P. & Lipp, H. P. A new computer program for detailed off-line analysis of swimming navigation in the Morris water maze. J. Neurosci. Methods 41, 65-74 (1992).

3. Ma, Y. et al. A three-dimensional digital atlas database of the adult C57BL/6 J mouse brain by magnetic resonance microscopy. Neurosci. 135,1203–1215 (2005).

4. Ma, Y. et al. In vivo 3D digital atlas database of the adult C57BL/6 J mouse brain by magnetic resonance microscopy. Front Neuroanat. 10.3389/neuro.05.001.2008. eCollection 2008.

5. Bhattacharya S, Herrera-Molina R, Sabanov V, Ahmed T, Iscru E, Stober F et al. Genetically Induced Retrograde Amnesia of Associative Memories After Neuroplastin Ablation. Biol Psychiatry 2016; Epub ahead of print 11 April 2016; doi: 10.1016/j.biopsych.2016.03.2107.

6. Herrera-Molina R, Sarto-Jackson I, Montenegro-Venegas C, Heine M, Smalla KH, Seidenbecher CI et al. Structure of excitatory synapses and GABAA receptor localization at inhibitory synapses are regulated by neuroplastin-65. J Biol Chem 2014; 289:8973-8988.

7. Bernstein, H. G. et al. The immunolocalization of the synaptic glycoprotein neuroplastin differs substantially between the human and the rodent brain. Brain Res. 1134, 107-112 (2007).

8. Gorski JA, Talley T, Qiu M, Puelles L, Rubenstein JL, Jones KR. Cortical excitatory neurons and glia, but not GABAergic neurons, are produced in the Emx1-expressing lineage. J Neurosci 2002; 22: 6309-6314.

9. Marin O, Rubenstein JL. A long, remarkable journey: tangential migration in the telencephalon. Nat Rev Neurosci 2001; 2: 780-790.

**Supplementary Table 1.**

Visual quantification of Np immunoreactivity in the human hippocampal formation

| Region | Layer | Signal intensity |
|---|---|---|
| **Fascia dentata** | I stratum moleculare | **++** |
| | II stratum granulosum | **++(+)** |
| | III stratum plexiforme | **+** |
| **Hilus** | I,II,III and IV layer | **(0)+** |
| **CA3** | Ia stratum moleculare | **0** |
| | Ib stratum lacunosum | **+** |
| | Ic stratum radiatum | **+** |
| | II stratum pyramidale | **+(+)** |
| | III stratum oriens | **(0)+** |
| **CA2** | Ia stratum moleculare | **0** |
| | Ib stratum lacunosum | **+** |
| | Ic stratum radiatum | **+** |
| | II stratum pyramidale | **+(+)** |
| | III stratum oriens | **0** |
| **CA1** | Ia stratum moleculare | **(0)+** |
| | Ib stratum lacunosum | **+** |
| | Ic stratum radiatum | **+(+)** |
| | II stratum pyramidale | **+(+)** |
| | III stratum oriens | **(0)+** |
| **Subiculum (includes prosubiculum subiculum, presubiculum, and parasubiculum)** | I stratum plexiforme | **0** |
| | II stratum pyramidale parvocellulare | **+** |
| | III stratum pyramidale magnocellulare | **0(+)** |
| | IV stratum pyramidale profundum | **(0)+** |
| | V stratum polymorfe | **-** |
| **Entorhinal cortex** | I stratum plexiforme | **0** |
| | II stratum stellatum | **+(+)** |
| | III stratum pyramidale superficiale | **+** |
| | Lamina dissecans | **0** |
| | IV stratum pyramidale profundum | **+(+)** |
| | V stratum pyramidale parvocellulare | **+(+)** |
| | VIa i VIb stratum polymorfe | **0(+)** |

**Supplementary Figure S1**

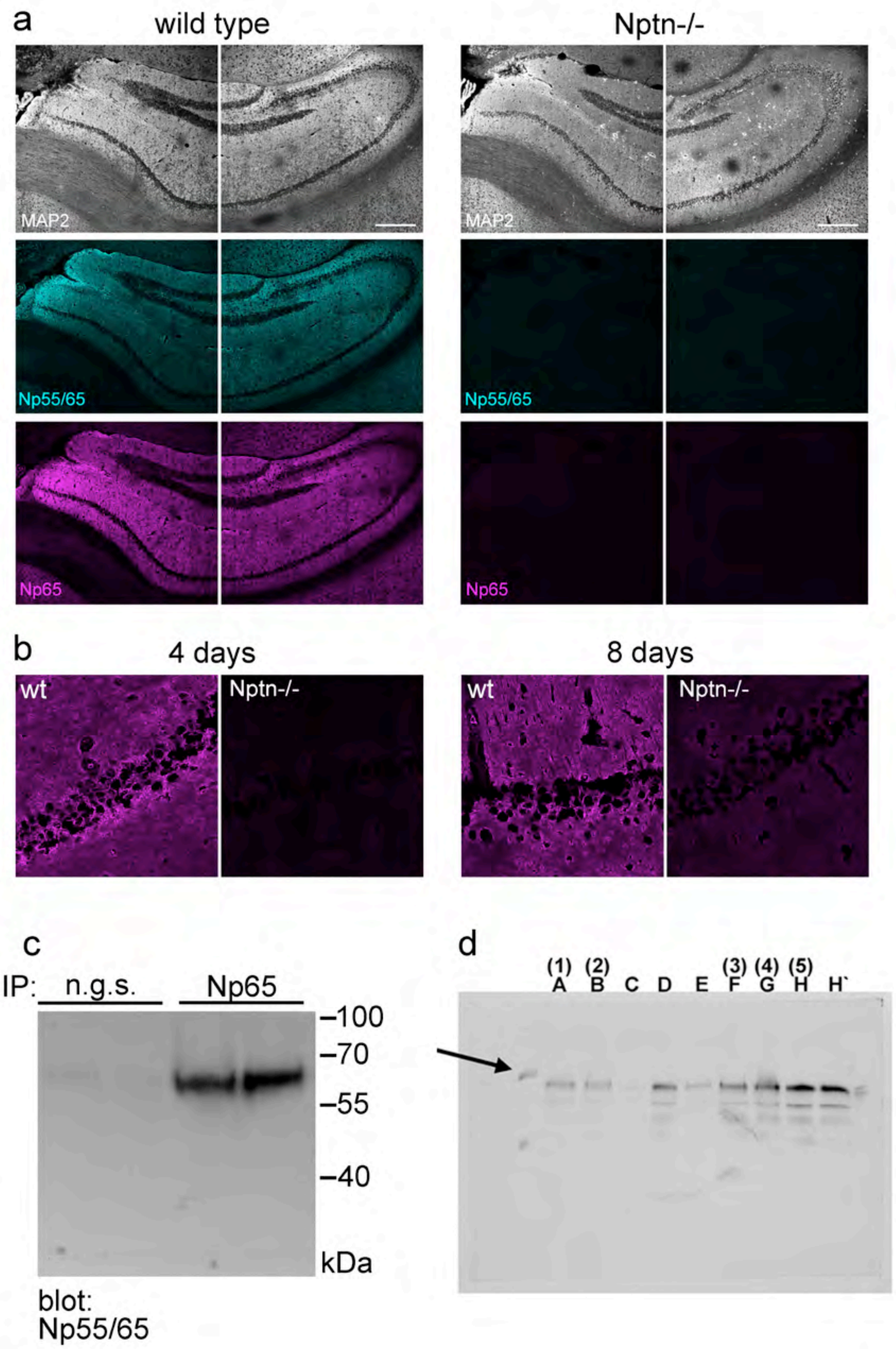

**Supplementary Figure S1. Immunodetection of mouse and human Np65.**

(**a**, **b**) Hippocampal slices were obtained from wild-type (wt) and neuroplastin-deficient (*Nptn*$^{-/-}$) brains after 4 or 8 days of post mortal delay mimicking the conditions the human hippocampal slices. Mouse slices were stained with anti-Np65 (magenta), pan-Np55/65 (cyan), and/or anti-MAP2 (grey) antibodies as indicated. Scale bar in **a** = 100µm. (**c**) Immunoprecipitation of Np65 from Triton-X100-soluble total extracts from two different samples of human hippocampal tissue was performed using the goat anti-Np65 antibody. Normal goat serum (n.g.s) was used to control the precipitation reaction. (d) Picture of the original of the blot displayed in Figure 1i showing Np55/65 in subcellular fractions from human brain cortex. A: total homogenate (corresponds to 1 in Fig. 1i), B: total membrane (corresponds to 2 in Fig. 1i), C: supernatant, D: microsomal fraction, E: myelin fraction, F: synaptic membrane (corresponds to 3 in Fig 1i), G: synaptic junction (corresponds to 4 in Fig 1i), H: post-synaptic density (corresponds to 5 in Fig 1i), H`: duplicate of post-synaptic density, arrow points to the molecular weight marker of 75kDa.

**Supplementary Figure S2**

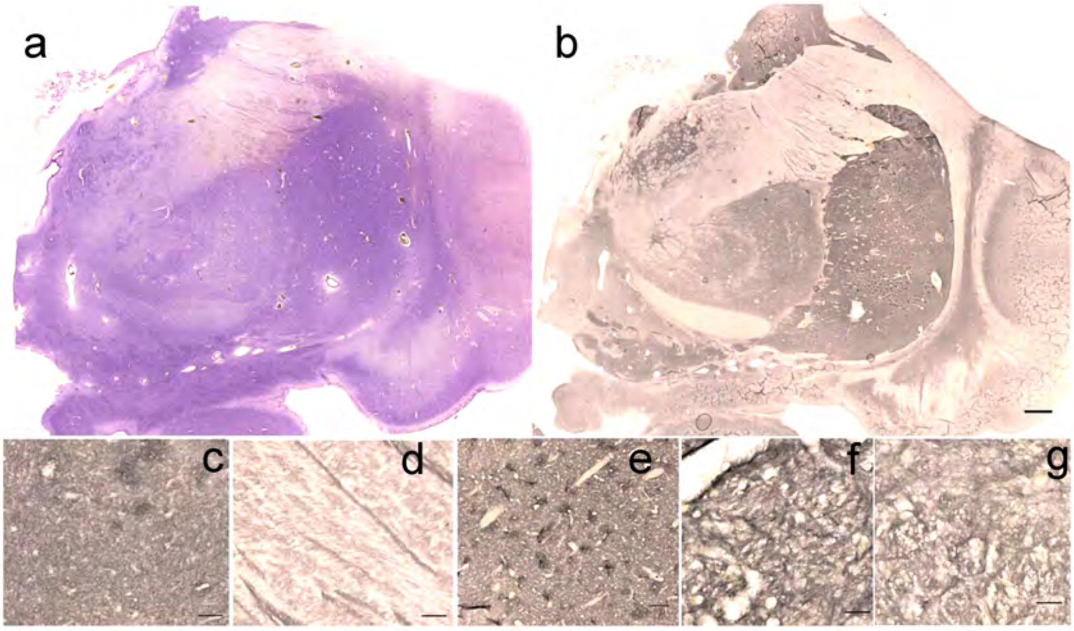


**Supplementary Figure S2. Np expression in the human striatum.** Np was detected using an anti-pan-hNp antibody (1:500, R&D Systems) and a DAB-based detection kit. (**a**) Nissl staining, (**b**) Np staining, (**c**) nucleus caudatus, (**d**) capsula interna, (**e**) putamen, (**f**) globus pallidus, pars externa, (**g**) globus pallidus, pars interna. Scale bar in **a** and **b** = 2mm; **c** and **d** = 100µm; **e** = 200µm; **f** and **g** = 50µm.

**Supplementary Figure S3**

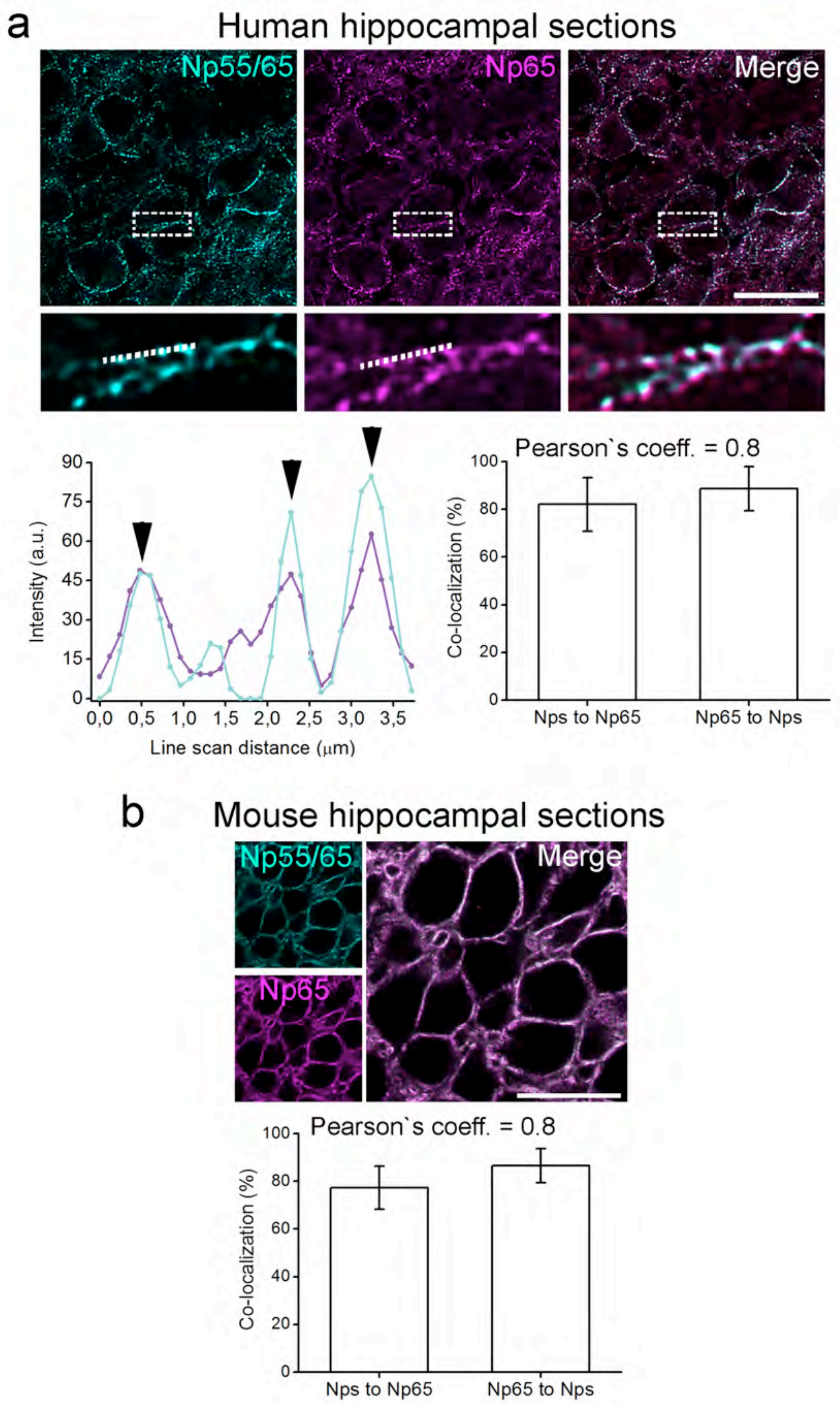

**Supplementary Figure S3. Np55 and Np65 are tightly co-localized in the cell plasma membrane of human and mouse neurons.** (**a**) Human and (**b**) Mouse hippocampal sections were immunostained with pan-anti-Np55/65 (cyan) and anti-Np65 (magenta) antibodies. Somata of granular cells in the dentate gyrus were photographed using a confocal microscope. Overlapping of both fluorescent signals (Merge) shows co-localization in white. (**a**) Digital magnifications from selected area (dotted squares) are displayed and the dotted lines (3.75 μm length) shown, as an example, scanned puncta used to confirm co-localization by line scanning the peak intensities of the signals (arrow heads, left graph). (**a** ,**b**) Co-localization quantification was performed considering a highly significant pixel-to-pixel correlation (Pearson's coefficient = 0.8). Scale bar in **a** = 20 μm, **b** = 25 μm.

**Supplementary Figure S4**

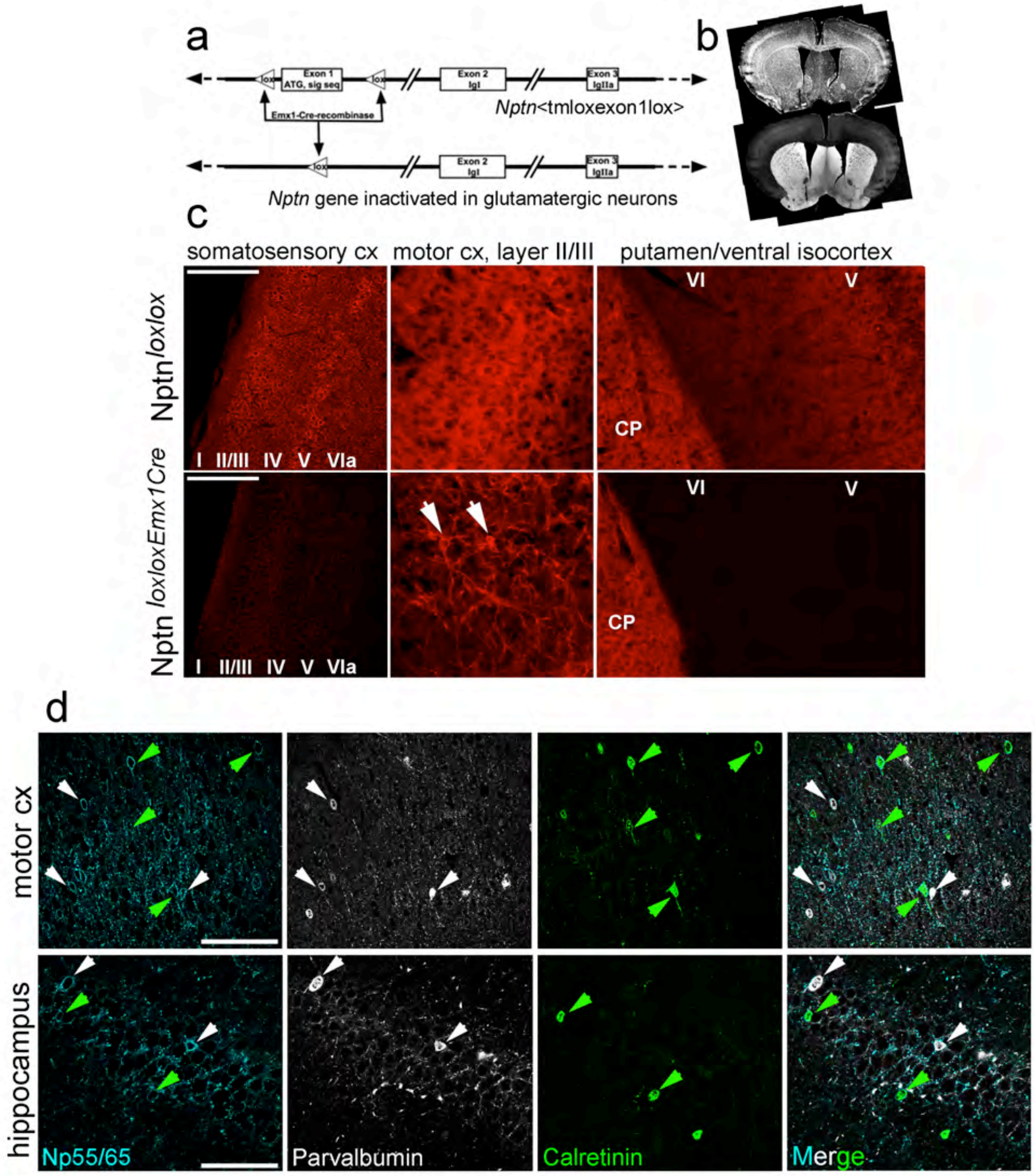

**Supplementary Figure S4. Elimination of Np in *Nptn*$^{loxloxEmx1Cre}$ mice.**

(**a**) Schematic diagram illustrating the strategy to ablate *Nptn* gene expression in Emx1-expressing glutamatergic neurons in the mouse hippocampus and cortex. (**b**) Fluorescent images of a *Nptn*$^{lox/loxEmx1Cre}$ brain section stained for DAPI (upper panel) and with anti-Np65 antibody (lower panel). (**c**) Somatosensory, motor, and ventral cortex and caudate putamen (CP) stained with anti-Np65-specific antibody (red). White arrows indicate interneurons that retain Np expression. (**d**) Confocal images of Np-positive interneurons (cyan) identified using anti-parvalbumin (grey) and anti-calretinin (green) antibodies in *Nptn*$^{lox/loxEmx1Cre}$ brain section. Scale bars in **c** = 240 μm, **d** = 40 μm.

**Supplementary Figure S5**

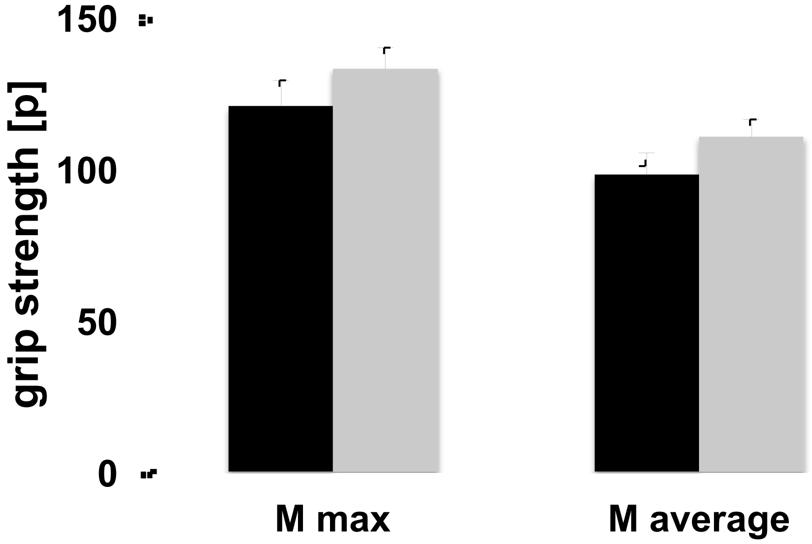


**Supplementary Figure S5. Grip Strength in *Nptn*$^{loxloxEmx1Cre}$ mice.**

Maximal (M max) and average (M average) grip strength of *Nptn*$^{loxloxEmx1Cre}$ mice (n=12, grey bars) were determined and compared to wild-type (*Nptn*$^{lox/lox}$ black bars, n=12) mice revealing similar physical strength (1-way ANOVA factor genotype, $P>0.05$).

**Supplementary Figure S6**

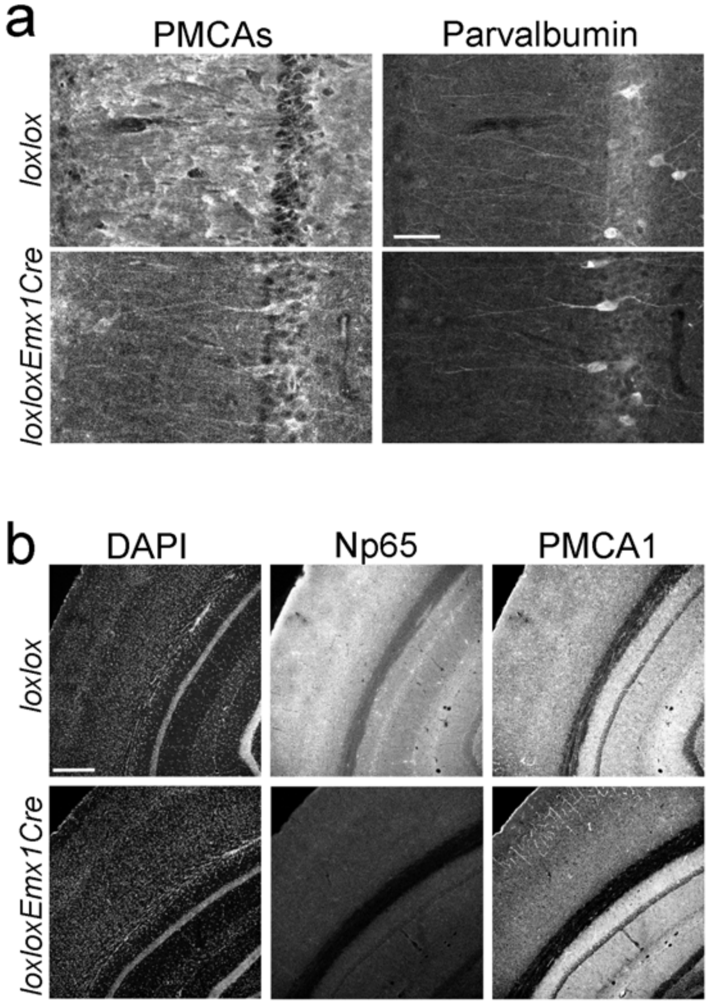


**Supplementary Figure S6. PMCA in interneurons and PMCA1 in *Nptn*$^{lox/loxEmx1Cre}$ mice.** (**a**) Confocal images of CA1 hippocampal area of *Nptn*$^{lox/lox}$ and *Nptn*$^{lox/loxEmx1Cre}$ mice stained for pan-PMCA (PMCAs) and parvalbumin. (**b**) Low magnification confocal images of cortex and hippocampus of *Nptn*$^{lox/lox}$ and *Nptn*$^{lox/loxEmx1Cre}$ mice stained for DAPI, Np65, and PMCA1. Scale bar in a = 50 µm and in b = 200 µm.
